# Strategic locations of white matter hyperintensities associated with the cognitive continuum among rural older Chinese adults: a population‐based study

**DOI:** 10.1002/alz70860_101824

**Published:** 2025-12-23

**Authors:** Chunyan Li, Jiafeng Wang, Ziwei Chen, Xiaodong Han, Cuicui Liu, Jie Lu, Huisi Zhang, Tao Gong, Yan Wang, Xiaoyu Wang, Qianqian Xie, Tingting Hou, yongxiang wang, Lin Cong, Lin Song, Yifeng Du, Chengxuan Qiu

**Affiliations:** ^1^ Shandong Provincial Hospital Affiliated to Shandong First Medical University, Jinan, Shandong, China; ^2^ Xuanwu Hospital, Capital Medical University, Beijing, Beijing, China; ^3^ Shandong Provincial Clinical Research Center for Neurological Diseases, Jinan, Shandong, China; ^4^ Shandong Provincial Hospital, Shandong University, Jinan, Shandong, China; ^5^ Institute of Brain Science and Brain‐Inspired Research, Shandong First Medical University & Shandong Academy of Medical Sciences, Jinan, Shandong, China; ^6^ Aging Research Center, Karolinska Institutet and Stockholm University, Stockholm, Sweden

## Abstract

**Background:**

The location of white matter hyperintensities (WMH) is crucial for cognitive phenotypes. We sought to identify strategic WMH locations associated with the cognitive continuum and subtypes of mild cognitive impairment (MCI).

**Method:**

This population‐based study used data derived from the MIND‐China MRI substudy (2018‐2020). Cognitive function was assessed using the neuropsychological test battery. Dementia, MCI, and subjective cognitive decline (SCD) were defined according to the international criteria. Total and tract‐specific WMH volumes were segmented and quantified by using the Lesion Prediction Algorithm. Data was analyzed using multinomial logistic regression models.

**Result:**

Of the 1253 participants (age ≥60 years), 28 were diagnosed with dementia, 286 with MCI, and 270 with SCD. WMH was highly prevalent in the periventricular and deep white matter regions, with bilateral anterior thalamic radiation (ATR) possessing the highest WMH volumes. Higher WMH volumes in bilateral corticospinal tract (CST) and right ATR were significantly related to various stages of cognitive impairment. Moreover, higher WMH in forceps minor and right superior longitudinal fasciculus was associated with dementia, WMH in left ATR and right inferior fronto‐occipital fasciculus was associated with MCI, and WMH in left superior longitudinal fasciculus was related to SCD. Higher WMH volumes in left ATR, right CST, and forceps minor were significantly associated with MCI among the non‐carriers, but not carriers, of the *APOE* ε4 allele (*p* for WMH volume×*APOE* ε4 allele interactions<0.05). In addition, WMH located in bilateral CST and left ATR was associated with amnestic MCI, while WMH in forceps major was associated with non‐amnestic MCI.

**Conclusion:**

CST and ATR represent strategic locations where high WMH is strongly associated with poor cognitive function across the cognitive continuum, emphasizing the importance of tract‐specific WMH in cognitive phenotypes.

